# Epigenetic Regulation of Corneal Epithelial Differentiation by TET2

**DOI:** 10.3390/ijms24032841

**Published:** 2023-02-02

**Authors:** Yuzuru Sasamoto, Siyuan Wu, Catherine A. A. Lee, Jason Y. Jiang, Bruce R. Ksander, Markus H. Frank, Natasha Y. Frank

**Affiliations:** 1 Division of Genetics, Brigham and Women’s Hospital, Boston, MA 02115, USA; 2 Transplant Research Program, Boston Children’s Hospital, Boston, MA 02115, USA; 3 Massachusetts Eye & Ear Infirmary, Schepens Eye Research Institute, Boston, MA 02114, USA; 4 Harvard Stem Cell Institute, Harvard University, Cambridge, MA 02138, USA; 5 Harvard Skin Disease Research Center, Department of Dermatology, Brigham and Women’s Hospital, Boston, MA 02115, USA; 6 School of Medical and Health Sciences, Edith Cowan University, Perth 6027, WA, Australia; 7 Department of Medicine, VA Boston Healthcare System, Boston, MA 02132, USA

**Keywords:** 5-hydroxymethylcytosine (5hmC), TET2, corneal epithelium, differentiation, genome-wide 5hmC analysis, KRT78, MYEOV, MAL

## Abstract

Epigenetic DNA modification by 5-hydroxymethylcytosine (5hmC), generated by the Ten-eleven translocation (TET) dioxygenases, regulates diverse biological functions in many organ tissues, including the mammalian eye. For example, 5hmC has been shown to be involved in epigenetic regulation of retinal gene expression. However, a functional role of 5hmC in corneal differentiation has not been investigated to date. Here, we examined 5hmC and TET function in the human cornea. We found 5hmC highly expressed in MUC16-positive terminally differentiated cells that also co-expressed the 5hmC-generating enzyme TET2. *TET2* knockdown (KD) in cultured corneal epithelial cells led to significant reductions of 5hmC peak distributions and resulted in transcriptional repression of molecular pathways involved in corneal differentiation, as evidenced by downregulation of MUC4, MUC16, and Keratin 12. Additionally, integrated *TET2* KD RNA-seq and genome-wide Reduced Representation Hydroxymethylation Profiling revealed novel epigenetically regulated genes expressed by terminally differentiated cells, including *KRT78*, *MYEOV*, and *MAL*. In aggregate, our findings reveal a novel function of TET2 in the epigenetic regulation of corneal epithelial gene expression and identify novel TET2-controlled genes expressed in differentiated corneal epithelial cells. These results point to potential roles for TET2 induction strategies to enhance treatment of corneal diseases associated with abnormal epithelial maturation.

## 1. Introduction

Corneal epithelial homeostasis is maintained by limbal stem cells (LSCs), which generate transit-amplifying cells migrating centripetally while giving rise to terminally differentiated apical cells [1]. These three main cell populations reside in distinct anatomical niches and can also be recognized by specific marker expression [2]. LSCs located at the basal epithelial layer in the outermost part of the cornea express ABCB5 and p63 [2,3,4,5,6,7,8,9,10]. Transit amplifying cells occupy the basal epithelial layer inside the limbal circle and are characterized by expression of ß-integrin and BCAM [11,12]. Terminally differentiated corneal epithelial cells are found in the apical layers of the central cornea, where they express MUC16 [13]. Despite a relative wealth of knowledge regarding the corneal epithelial hierarchy and transcriptional control of corneal differentiation, the role of epigenetic regulation in corneal gene expression is not well understood.

The significance of epigenetic DNA modifications in eye development and disease has been increasingly recognized over the last decade [14]. DNA methylation at the fifth position of cytosine (5mC) serves as a critical epigenetic modifier of gene expression [15]. Abnormal 5mC levels have been reported in association with aberrant wound healing [16] and Fuchs corneal dystrophy [17]. The Ten-eleven translocation (TET) family of dioxygenases, TET1, TET2, and TET3, modulate DNA methylation patterns through conversion of 5mC to 5-hydroxymethylcytosine (5hmC) [18]. Studies have shown that 5hmC, in addition to being an intermediate of 5mC oxidation, also serves as a stable epigenetic mark with regulatory functions [19]. 5hmC enrichment was reported at genes involved in diverse biological functions, including development, tumorigenesis, stemness, and differentiation [20,21]. However, the role of 5hmC epigenetic modification in corneal differentiation has not been examined to date.

During vertebrate evolution, TET2 diverged from TET1 and TET2 members of the TET family as a result of gene fission, which split the CpG DNA binding CXXC domain into a separate IDAX protein encoded by the CXXC4 gene transcribed in the opposite direction [22]. IDAX, which also serves as an inhibitor of Wnt signaling [23], downregulates TET2 protein expression [22]. In the eye, TET2 was found to play an important role in retinal neurogenesis [24].

Here, we uncover a novel function of TET2 in the regulation of corneal epithelial gene expression through 5hmC modification. In addition, using genome-wide 5hmC profiling correlated with RNA-seq of *TET2* KD cells, we identify novel epigenetically regulated genes involved in corneal differentiation.

## 2. Results

### 2.1. 5hmC Marks Quiescent Cells in the Limbus

**Figure 1:**
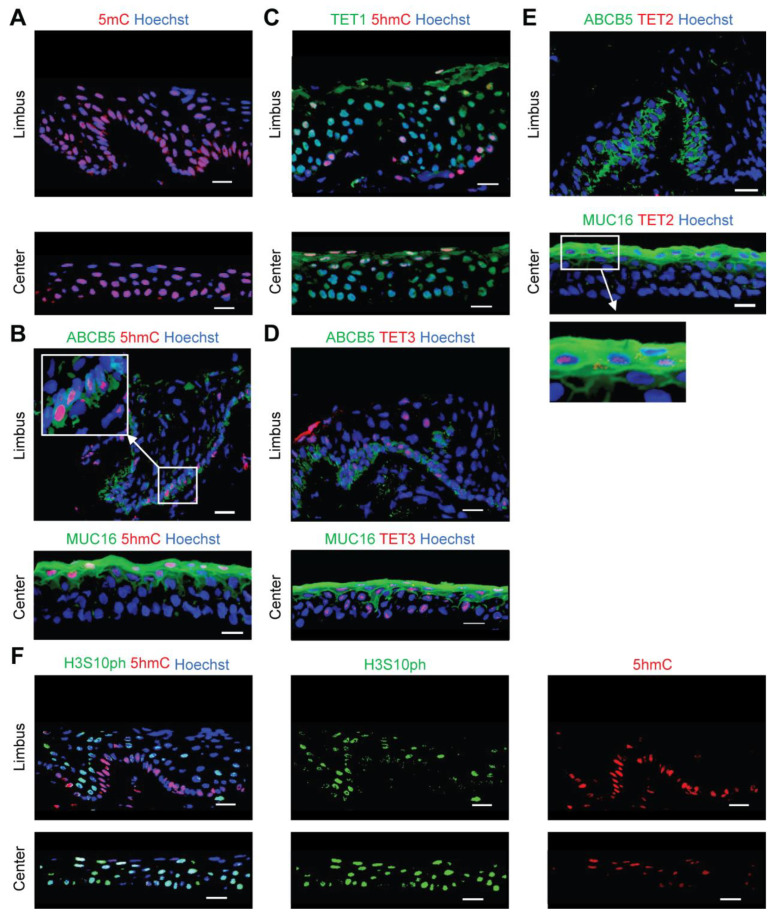
Characterization of 5mC and 5hmC expression in the human cornea. Representative immunostaining analyses of: (**A**) 5mC expression in the limbus (**top**) and central cornea (**bottom**), (**B**) ABCB5 and 5hmC co-expression in the limbus (**top**), and MUC16 and 5hmC co-expression in the central cornea (**bottom**), (**C**) TET1 and 5hmC co-staining in the limbus (**top**) and central cornea (**bottom**), (**D**) Co-expression of ABCB5 and TET3 in the limbus (**top**), and co-staining of MUC16 and TET3 in the central cornea (**bottom**), (**E**) ABCB5 and TET2 co-staining in the limbus (**top**), and MUC16 and TET2 co-expression in the central cornea (**bottom**), (**F**) Co-staining of H3S10ph and 5hmC in the limbus (**top**) and the central cornea (**bottom**). Nuclei stained with Hoechst 33342 (blue), n = 3. Scale bar, 20 µm.

We performed in situ 5mC and 5hmC immunostaining to examine the epigenetic landscape of the human cornea. We found nearly ubiquitous expression of 5mC (Figure 1A). However, 5hmC was mainly detected in ABCB5-positive LSCs located in the basal limbus and in MUC16-positive apical differentiated central corneal cells (Figure 1B). TET1 and TET3 were expressed throughout all corneal layers in the limbus and central cornea (Figure 1C,D). In contrast, TET2 expression was restricted to the MUC16-positive apical differentiated central corneal epithelium (Figure 1E), suggesting a specific function of TET2 in the maintenance of enhanced 5hmC levels during corneal differentiation. Notably, 5hmC-positive cells in the limbus expressed low levels of phosphorylation of serine 10 on histone H3 (H3S10ph) [25] (Figure 1F), pointing to an association of 5hmC expression with cellular quiescence.

### 2.2. TET2 Regulates the Expression of Corneal Differentiation Genes

**Figure 2:**
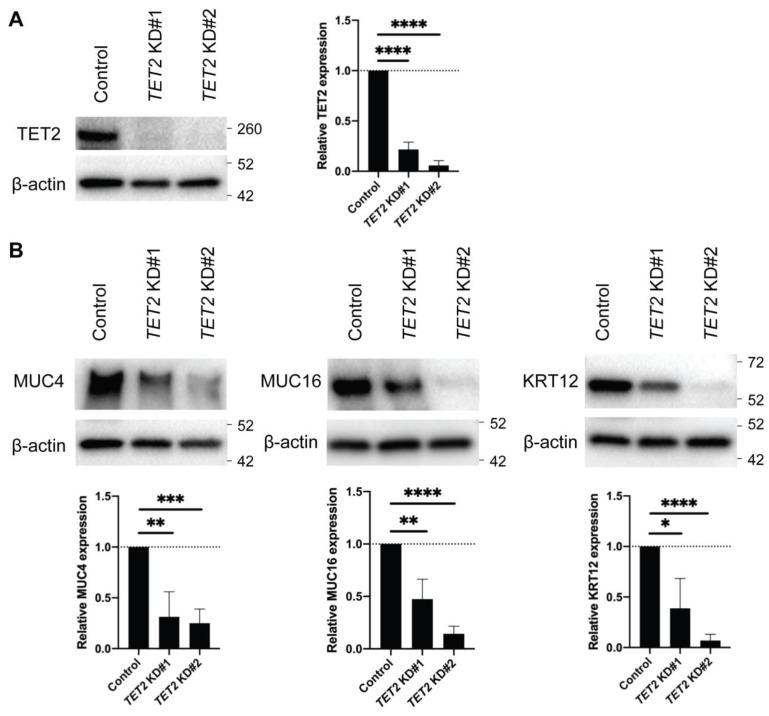
Expression of corneal differentiation genes in *TET2* KD cultured corneal epithelial cells. (**A**) **Left**, Western blot analyses of TET2 protein expression in *TET2* KD cells. **Right**, bar graphs represent the quantitative analyses of TET2 protein expression (*TET2* KD#1: 21.6 ± 7.2%, *TET2* ± KD#2: 5.8 ± 4.7%, n = 5; **** *p* < 0.0001). Data were analyzed using a Tukey’s multiple comparisons test. (**B**) **Top**, Western blot analyses of MUC4, MUC16 and KRT12 expression in *TET2* KD cells. **Bottom**, bar graphs represent the quantitative analyses of expressions of MUC4, MUC16, and KRT12 protein (*TET2* KD#1: 31.3 ± 24.7%, *TET2* ± KD#2: 25.1 ± 13.9% for MUC4, *TET2* KD#1: 47.5 ± 19.0%, *TET2* ± KD#2: 14.3 ± 7.3% for MUC16, *TET2* KD#1: 38.8 ± 29.5%, *TET2* ± KD#2: 7.0 ± 6.0% for KRT12; n = 5, * *p* < 0.05, ** *p* < 0.01, *** *p* < 0.001 and **** *p* < 0.0001). Data were analyzed using a Tukey’s multiple comparisons test.

**Figure 3:**
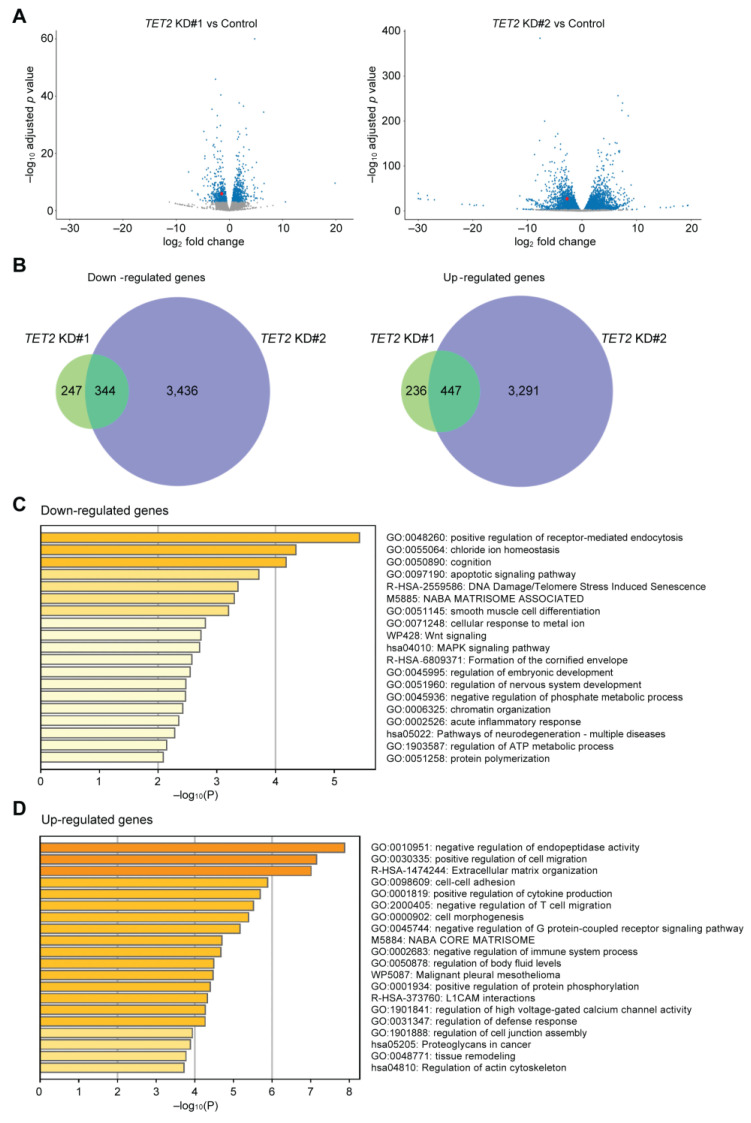
Characterization of TET2-regulated pathways in corneal epithelial cells. (**A**) Volcano plots depicting mRNA expression levels in *TET2* KD#1 (**left**) and *TET2* KD#2 (**right**) cultures. Blue dots represent differentially expressed genes (DEGs) selected as described in the methods. TET2 expression is depicted with red dots. (**B**) Venn diagrams of the overlapping downregulated genes (**left**) and upregulated genes (**right**) in *TET2* KD#1 and *TET2* KD#2 cultures. Metascape enrichment analysis of the downregulated (**C**) and upregulated (**D**) DEGs shared by both *TET2* KD#1 and *TET2* KD#2 cultures. All experiments were repeated n = 3 times.

To investigate the role of TET2 in corneal epithelial differentiation, we performed *TET2* knockdown (KD) in cultured corneal epithelial cells by two distinct siRNAs, designated as *TET2* KD#1 and *TET2* KD#2 (Figure 2A). Both *TET2* KD#1 and *TET2* KD#2 led to significant downregulation of the corneal epithelial differentiation markers MUC4, MUC16 [13] and Keratin 12 (KRT12) [26] (Figure 2B). RNA-seq analyses (Figure 3A) identified 344 downregulated and 447 upregulated transcripts shared by both *TET2* KD#1 and *TET2* KD#2 cultures (Figure 3B, Supplemental Tables S1 and S2). Metascape enrichment analyses of these differentially expressed genes (DEGs) revealed downregulation of terminal differentiation-related pathways such as the apoptotic signaling pathway and the formation of the cornified envelope pathway (Figure 3C), and induction of basal epithelial cell-related pathways such as positive regulation of cell migration and cell–cell adhesion (Figure 3D).

### 2.3. Identification of Genes Regulated by TET2 through 5hmC Modification

**Figure 4:**
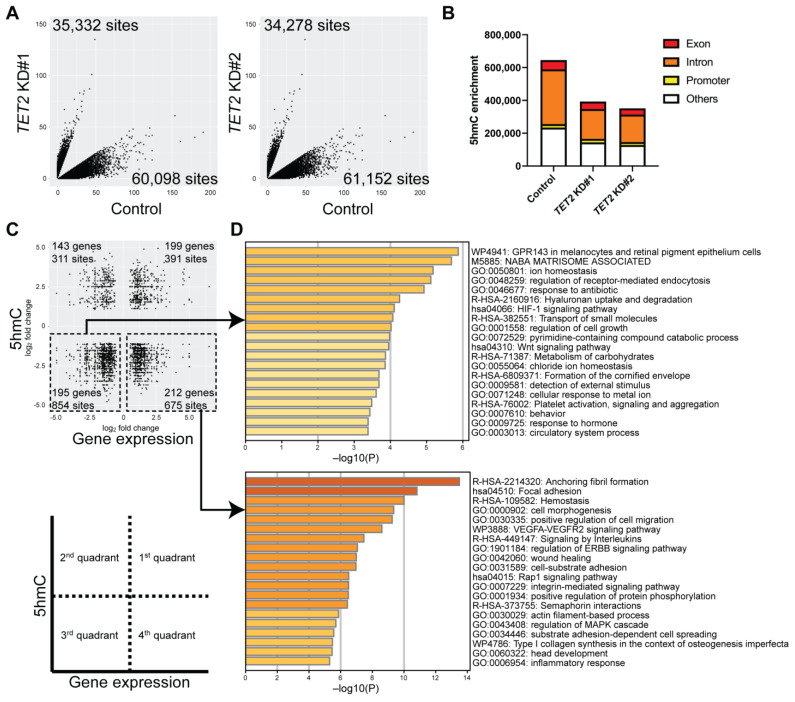
Identification of epigenetically regulated pathways through integration of RNA-seq and Reduced Representation Hydroxymethylation Profiling (RRHP) analyses of *TET2* KD cells. (**A**) Scatter plots of 5hmC enrichment in *TET2* KD#1 (**left**) and *TET2* KD#2 (**right**) cultures. (**B**) Bar graphs represent the distribution of 5hmC site numbers in exon, intron, and promoter regions. (**C**) Scatter plots depict the correlation between the DEGs (*x*-axis) and corresponding differentially enriched 5hmC levels (*y*-axis) shared between *TET2* KD#1 and *TET2* KD#2 cultures selected as described in methods. (**D**) Metascape enrichment analysis of the downregulated (**top**) and upregulated (**bottom**) DEGs with reduced 5hmC levels.

To identify TET2-regulated epigenetic loci, we performed Reduced Representation Hydroxymethylation Profiling (RRHP) of *TET2 KD* cells compared to controls. We observed a significant reduction of 5hmC site numbers in various exon, intron, and promoter regions (*p* = 0.0260) (Figure 4A,B). Next, we correlated the changes in gene expression with changes in their respective 5hmC levels (Figure 4C). Overall, we observed 1329 reduced 5hmC peaks in 407 differentially expressed genes in *TET2* KD cells (Figure 4C). Metascape analyses of the downregulated genes revealed enrichment of specific pathways involved in Wnt signaling and formation of the cornified envelope. In contrast, upregulated genes were involved in focal adhesion, cell morphogenesis, and positive regulation of cell migration pathways (Figure 4D). Of note, we found that the corneal differentiation markers *MUC4* and *MUC16* found downregulated in *TET2* KD cells also exhibited reduced 5hmC peaks in their exon and intron regions (Table 1).

### 2.4. Identification of Novel Corneal Differentiation Genes Epigenetically Regulated by TET2

**Figure 5:**
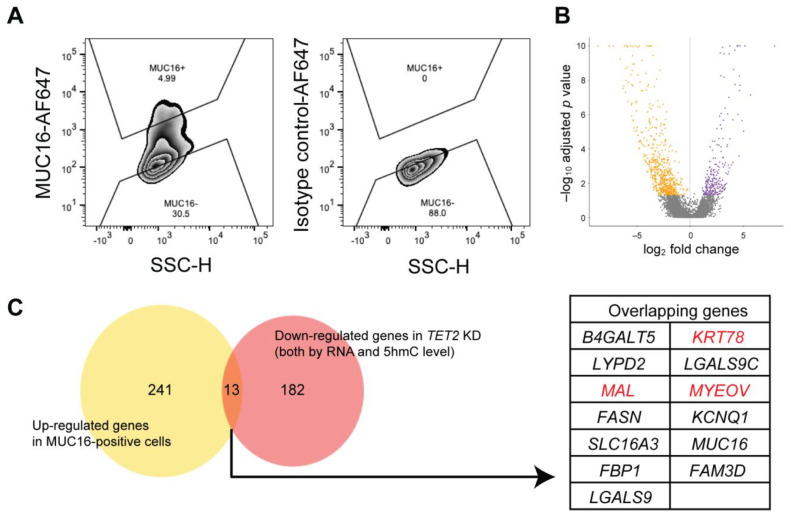
Identification of TET2-regulated genes enriched in differentiated corneal cells. (**A**) Representative flow cytometry analyses of MUC16 expression in central corneal epithelial cells. SSC, side scatter; H, height: AF647, Alexa Fluor 647. (**B**) Volcano plot depicting DEGs between flow cytometrically sorted MUC16-positive and MUC16-negative cells. Orange dots depict the genes enriched in MUC16-negative cells. Purple dots represent the genes enriched in MUC16-positive cells. Genes were selected as described in the methods. (**C**) **Left**, Venn diagram of the genes upregulated in MUC16-positive cells and the downregulated genes in *TET2* KD cells. **Right**, the table shows the list of 13 overlapping genes. KRT78, MAL, and MYEOV highlighted in red were selected for functional analyses, as described in Figure 6.

**Figure 6:**
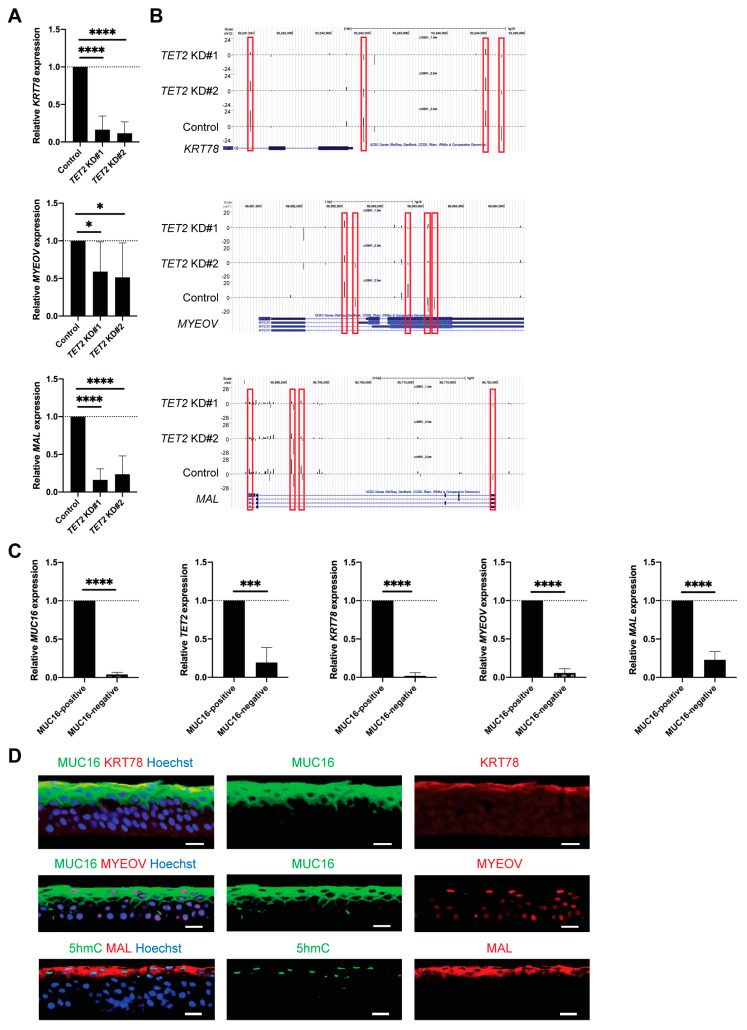
Identification of KRT78, MYEOV, and MAL as epigenetically regulated corneal differentiation genes. (**A**) Bar graphs represent relative *KRT78*, *MYEOV* and *MAL* mRNA expression in *TET2* KD cells (*TET2* KD#1: 16.4 ± 18.2%, *TET2* ± KD#2: 11.5 ± 15.3% for *KRT78*, *TET2* KD#1: 59.1 ± 39.5%, *TET2* ± KD#2: 51.4 ± 45.9% for *MYEOV*, *TET2* KD#1: 16.2 ± 14.5%, *TET2* ± KD#2: 23.7 ± 24.3% for *MAL*; n = 8, * *p* < 0.05 and **** *p* < 0.0001). Data were analyzed using a Tukey’s multiple comparisons test. (**B**) UCSC genome browser depiction of reduced 5hmC enrichment peaks in the *KRT78*, *MYEOV* and *MAL* genomic regions of *TET2* KD cells. (**C**) Bar graphs represent *MUC16*, *TET2*, *KRT78*, *MYEOV*, and *MAL* mRNA expression in sorted MUC16-positive and MUC16-negative cells (relative expression in MUC16-negative cells: 3.9 ± 2.8% for *MUC16*, 19.3 ± 19.6% for *TET2*, 1.9 ± 4.2% for *KRT78*, 5.6 ± 5.5% for *MYEOV*, 23.0 ± 10.8% for *MAL*; n = 5, *** *p* < 0.001, **** *p* < 0.0001). Data were analyzed using a paired *t*-test. (**D**) Representative immunostaining images of MUC16 and KRT78 (**top**), MUC16 and MYEOV (**middle**), and 5hmC and MAL (**bottom**) in the central corneal epithelium. Hoechst 33342 was used for the nuclei staining. n = 3. Scale bar, 20 μm. The red frames depict the 5hmC peaks reduced in TET2 KD cells.

Based on the high TET2 and 5hmC levels in the apical corneal epithelial layers, we hypothesized that TET2 is involved in the epigenetic regulation of genes associated with corneal differentiation. We used cell-surface expressed protein MUC16 to prospectively isolate differentiated corneal epithelial cells [27] and performed RNA-seq analysis of sorted MUC16-positive and MUC16-negative cells isolated from n = 3 human donor corneas to characterize the transcripts associated with corneal differentiation (Figure 5A,B). Next, we compared the genes enriched in MUC16-positive cells to the genes downregulated by *TET2* KD (Figure 5C). This comparison identified 13 transcripts, seven of which, i.e., *KRT78*, *MYEOV*, *MAL*, *LYPD2*, *LGALS9C*, *B4GALT5*, and *LGALS9*, also had reduced 5hmC levels. Since the association of *LYPD2*, *LGALS9C*, *B4GALT5*, and *LGALS9* with corneal epithelial differentiation has been reported previously [27,28,29], we focused our further studies on the characterization of *KRT78*, *MYEOV*, and *MAL*. Reduced expression of KRT78, MYEOV, and MAL in *TET2* KD cells was validated by RT-PCR and Western blot analyses (Figure 6A and Supplementary Figure S1) and attenuated 5hmC peaks were detected by RRHP in their corresponding genomic regions (Figure 6B). Moreover, we found significant overexpression of *KRT78*, *MYEOV*, and *MAL*, as well as of *MUC16* and *TET2* transcripts, in sorted MUC16-positive cells by RT-PCR (Figure 6C). In situ KRT78, MYEOV, and MAL protein expression was detected exclusively in the apical corneal epithelial layers, where they co-expressed MUC16 or 5hmC (Figure 6D). Thus, our findings identify KRT78, MYEOV, and MAL as novel epigenetically regulated markers of corneal epithelial differentiation.

## 3. Discussion

In the current study, we reveal a novel function of TET2 in epigenetic regulation of corneal epithelial gene expression. Using in situ immunofluorescence analyses, RNA-seq and RRHP profiling, we identified TET2-controlled genes expressed by differentiated corneal epithelial cells. Our findings point to a potential role of TET2-inducing strategies for the treatment of corneal diseases associated with abnormal epithelial maturation.

Using in situ immunostaining, we discovered a dichotomy of 5hmC distribution in the human cornea. Increased 5hmC expression was detected in ABCB5-positive LSCs and MUC16-positive terminally differentiated cells, while the majority of transit amplifying and differentiating MUC16-negative cells exhibited low 5hmC levels. These findings are in line with previous observations of high 5hmC levels in stem cells such as embryonic stem cells (ESCs) [30,31] and hematopoietic stem cells [32] and in terminally differentiated cells such as differentiated epithelial cells [33], but reduced 5hmC levels in proliferative cells such as basal cells in the small intestine, the skin epithelium [33], and in neural progenitor cells [34]. High 5hmC levels observed upon differentiation are thought to be associated with a “poised” chromatin configuration, which activates transcription in response to environmental stimuli, whereas in undifferentiated ESCs high 5hmC levels are correlated with transcriptional repression regulated by TET1 [31].

In our study of the cornea, we observed high TET1, TET3, and 5hmC levels in ABCB5-positive LSCs, which could relate to their undifferentiated stem cell phenotype. While we also detected continuing TET1 and TET3 expression in early and late transit amplifying cells and differentiating suprabasal cells, their 5hmC levels were low or undetectable. This finding can be explained by continuing oxidation of the 5hmC by TET1 and TET3 in these cells and their proliferation. TET2 was specifically localized to terminally differentiated MUC16-positive cells where it was co-expressed with 5hmC. Intriguingly, while *TET2* KD led to significant reduction of 5hmC peaks, it was associated with transcriptional repression of pathways involved in corneal differentiation along with activation of basal epithelial cell-related pathways such as positive regulation of cell migration and cell-cell adhesion. Additional future studies at the single cell level might serve to further dissect such divergent effects of TETs on epigenetic regulation of gene expression in the cornea. 

Consistent with the essential role of TET2 in corneal epithelial differentiation, *TET2* KD was associated with significant downregulation of distinct pathways involved in Wnt signaling and in the formation of the cornified envelope, with corresponding reduction of 5hmC levels. *TET2* KD hereby reduced expression of the established corneal differentiation markers MUC4, MUC16, and KRT12. Furthermore, integration of *TET2* KD RNA-seq and genome-wide 5hmC analyses revealed additional epigenetically regulated corneal differentiation genes, 13 of which were also highly expressed in MUC16-positive cells. Three of those genes, i.e., *KRT78*, *MYEOV*, and *MAL,* had not previously been specifically described in the cornea. Our flow cytometry and immunostaining analyses now demonstrate, for the first time, their high expression among apical differentiated corneal epithelial cells, suggesting a potential role of these genes in corneal epithelial formation.

Corneal epithelial maturation plays a critical role in the formation of the intact and clear ocular surface. Abnormal corneal epithelial differentiation has been observed in several ocular surface diseases, including dry eye disease [35], diabetes mellitus-related compromised barrier function [36], and enhanced epithelial fragility [37]. The cornea barrier function is highly dependent on the formation of tight epithelial junctions, as well as mucins, which protect the cornea from external allergens and pathogens [38,39]. Therefore, our findings implicating TET2 in epigenetic regulation of corneal epithelial gene expression point to potential therapeutic roles for TET2-inducing approaches for the treatment of ocular surface diseases.

In sum, our study reveals an essential novel role of TET2 in epigenetic control of corneal epithelial differentiation and discovers novel epigenetically regulated genes expressed by terminally differentiated cells. Therefore, our results raise the possibility of therapeutic potential of TET2-inducing therapies for the treatment of eye diseases associated with abnormal epithelial maturation.

## 4. Methods

### 4.1. Human Tissues

Human whole globes and corneal tissues were obtained from the Saving Sight eye bank, Kansas City, MO, and the CorneaGen eye bank, Seattle, WA, under Institutional Review Board (IRB)-approved protocols.

### 4.2. Tissue Processing

For immunostaining, whole eye globes were processed as follows: One group was fixed with 10% neutral buffered formalin (Fisher Scientific, Pittsburgh, PA, USA) at 4 °C overnight and submerged into 70% ethanol. Subsequently, the tissues were paraffin-embedded at the Brigham and Women’s Hospital’s (BWH) Pathology Core. Another group was embedded in TissueTek^®^ O.C.T Compound (Sakura, Tokyo, Japan) and kept frozen at −80 °C. For cell culture and flow cytometry, 8mm diameter central cornea sections were separated from the remaining corneal tissues by a disposable biopsy punch (Integra LifeSciences, Plainsboro, NJ, USA). After the mechanical removal of corneal endothelial cells, limbal and central corneal tissues were incubated with PluriSTEM Dispase II Solution (MilliporeSigma, Burlington, MA, USA) at 37 °C for 1 h. Limbal and central corneal epithelial cells were scraped and incubated with TrypLE Express Enzyme (Thermo Fisher Scientific, Waltham, MA, USA) at 37 °C for 30 min. For corneal epithelial cell culture, dissociated limbal epithelial cells were maintained in DMEM/F12 medium (Thermo Fisher Scientific) supplemented with 10 ng/mL keratinocyte growth factor (KGF) (PeproTech, Rocky Hill, NJ, USA), 10 μM Y-27632 (Tocris Bioscience, Bristol, UK) and B-27 Supplement (Thermo Fisher Scientific) [40].

### 4.3. Immunostaining

Formalin-fixed paraffin-embedded sections were subjected to deparaffinization and antigen retrieval steps before immunostaining as described [12]. The tissue sections were permeabilized with Triton-X (MilliporeSigma) containing buffer and blocked with 5–10% normal donkey or goat serum (Jackson ImmunoResearch Laboratories, West Grove, PA) or 1% bovine serum albumin (BSA) (MilliporeSigma) containing buffer. The fresh frozen sections were denatured with 2N-HCl (VWR, Radnor, PA, USA) at room temperature for 15 min and incubated with 100 mM Tris-HCl (pH 8.0) (Boston BioProducts, Milford, MA, USA) at room temperature for 10 min before blocking. Blocked sections were incubated with primary antibodies at 4 °C overnight. Primary antibodies used in the study were mouse anti-5mC polyclonal antibody (pAb) (1:5000, Active Motif, Carlsbad, CA, USA), rabbit anti-5hmC pAb (1:5000, Active Motif), mouse anti-ABCB5 monoclonal antibody (mAb) (clone 3C2-1D12) (10 μg/mL, [41]), mouse anti-MUC16 mAb (1:400, Abcam, Cambridge, UK), mouse anti-TET1 mAb (1:200, GeneTex, Irvine, CA, USA), rabbit anti-TET2 pAb (1:200, GeneTex), rabbit anti-TET3 pAb (1:400, GeneTex), mouse anti-H3S10ph mAb (1:250, GeneTex), rabbit anti-KRT78 pAb (1:200, MyBiosource, San Diego, CA, USA), rabbit anti-MYEOV pAb (1:100, Atlas Antibodies, Bromma, Sweden), and mouse anti-MAL mAb (1:200, Santa Cruz Biotechnology, Santa Cruz, CA, USA). Sections were incubated with Alexa fluor 488 or 568-conjugated secondary antibodies (1:400, Abcam) at room temperature for 1 h and with Hoechst 33342 (1:400, Thermo Fisher Scientific) at room temperature for 10 min. The image capture was performed using a C2+ confocal microscope (Nikon, Tokyo, Japan) and analyses were performed using NIS-Elements AR v4.30.01 (Nikon).

### 4.4. RNA Interference

Gene knockdown (KD) experiments were performed as reported previously [12,42]. Briefly, transfection of cultured corneal epithelial cells was performed using Lipofectamine™ RNAiMAX Transfection Reagent (Thermo Fisher Scientific). *Silencer*^TM^ Select siRNAs (Thermo Fisher Scientific) used were *Silencer*™ Select Negative Control No.1 siRNA and *TET2* siRNAs (*TET2* KD#1 [s29441] and *TET2* KD#2 [s29442]). siRNAs were transfected on days 0, 2, and 4 and the cells were harvested on day 7.

### 4.5. Western Blot

Cell lysis and protein extraction were performed using RIPA buffer (Cell Signaling Technology, Danvers, MA, USA) supplemented with cOmplete™ Protease Inhibitor Cocktail (MilliporeSigma). The lysates were mixed with SDS-sample buffer (Boston BioProducts) and 2-mercaptoethanol (MilliporeSigma) and denatured at 95 °C for 10 min. The proteins separated by SDS-PAGE gel electrophoresis (Bio-rad, Hercules, CA, USA) were transferred onto PVDF blotting membranes (GE Healthcare Life Sciences, Marlborough, MA, USA). After blocking with 5% blotting-grade blocker (Bio-Rad) containing buffer at room temperature for 1 h, the membranes were incubated with primary antibodies diluted in 2.5% blocking buffer at 4 °C overnight. Primary antibodies used were rabbit anti-β-actin pAb (1:1000, Cell Signaling Technology), rabbit anti-TET2 pAb (1:2000, GeneTex), rabbit anti-MUC4 pAb (1:1000, Abcam), rabbit anti-MUC16 mAb (1:10,000, Abcam), rabbit anti-KRT12 mAb (1:5000, Abcam), rabbit anti-KRT78 pAb (1:500, MyBiosource), and rabbit anti-MYEOV pAb (1:1000, MyBiosource). The membranes were subsequently incubated with HRP-conjugated secondary antibodies (1:1000, Cell Signaling Technology) at room temperature for 1 h and signals were developed by Western Lightning Plus-ECL (PerkinElmer, Waltham, MA, USA) or SuperSignal^TM^ West Femto Maximum Sensitivity Substrate (Thermo Fisher Scientific). The images were acquired using the ChemiDoc MP Imaging System (Bio-Rad). Relative protein expression levels against β-actin expression were calculated using Image Lab software v5.2.1 (Bio-Rad).

### 4.6. RNA-Seq

Total RNA was extracted either by AllPrep DNA/RNA Mini Kit (QIAGEN, Hilden, Germany) or RNeasy Plus Mini Kit (QIAGEN). After the removal of contaminating genomic DNA by a DNA-free™ DNA Removal Kit (Thermo Fisher Scientific), mRNA libraries were generated by SMART-Seq^®^ v4 Ultra^®^ Low Input RNA Kit for Sequencing (Clontech, Mountain View, CA, USA). Sequencing was carried out by the Illumina NextSeq 500 Platform (Single-end 75 bp) (Illumina, San Diego, CA, USA) at the Molecular Biology Core Facility (MBCF) of the Dana–Farber Cancer Institute. RNA-seq counts were generated by Salmon [43]. Differential gene expression (DEG) analyses were performed using DESeq2 [44]. DEGs with adjusted *p* values < 0.05 were used for further analyses. The pathway enrichment in DEGs was determined using the Metascape software (http://metascape.org, accessed on 14 February 2022) [45]. DEGs are listed in Supplementary Tables S1 and S2 for *TET2* KD and Supplementary Tables S5 and S6 for MUC16-positive vs. MUC16-negative.

### 4.7. Genome-Wide 5hmC Analysis

Genomic DNA was extracted using an AllPrep DNA/RNA Mini Kit (QIAGEN). Genome-wide 5hmC analysis (Reduced Representation Hydroxymethylation Profiling: RRHP) was performed by Zymo Research (Irvine, CA, USA) [46]. The 5hmC enrichment data from the control sample were compared against *TET2* knockdown samples. Read data from the top (“+”) and bottom (“−”) strands were considered separately to account for asymmetric 5hmC modification along a CpG palindrome of double-stranded DNA. A log_2_ fold change was computed for each comparison. The following criteria were applied to define differential 5hmC peaks: at least one sample must have coverage > 4 and at least one comparison must show log_2_ fold change > 1. Genes that were differentially expressed at the RNA level and differentially enriched at the 5hmC level were compiled. For each siRNA condition (*TET2* KD#1 and *TET2* KD#2) and each gene region (exon, intron, and promoter), RNA-seq log_2_ fold change was plotted on the *x*-axis against 5hmC log_2_ fold change on the *y*-axis. Complete RRHP data are available in Supplementary Tables S3 and S4.

### 4.8. Quantitative Reverse Transcription PCR (qRT-PCR)

Total RNA was extracted using either an AllPrep DNA/RNA Mini Kit or an RNeasy Plus Mini Kit (QIAGEN). After the removal of contaminating genomic DNA by a DNA-free™ DNA Removal Kit (Thermo Fisher Scientific) from extracted RNA, cDNA synthesis was performed using a High-Capacity cDNA Reverse Transcription Kit (Thermo Fisher Scientific). qPCR was conducted with TaqMan^TM^ Gene Expression Assay probes (Thermo Fisher Scientific) and TaqMan^TM^ Fast Universal PCR Master Mix (Thermo Fisher Scientific). The TaqMan^TM^ probes used were *GAPDH* (Hs99999905_m1), *KRT78* (Hs00542779_m1), *MYEOV* (Hs00371084_m1), *MAL* (Hs00242748_m1), *MUC16* (Hs01065175_m1), and *TET2* (Hs00325999_m1). The PCR cycling condition was 95 °C for 20 s and 50 cycles of [95 °C/1 s; 60 °C/20 s] using a StepOnePlus™ Real-Time PCR System (Thermo Fisher Scientific). Calculation of ΔΔCt values was performed using *GAPDH* as a reference gene.

### 4.9. Flow Cytometric Analyses

Dissociated central corneal epithelial cells were stained with 1.0 μg/mL mouse anti-MUC16 mAb (Abcam) conjugated with Alexa Fluor 647 (Thermo Fisher Scientific) on ice for 30 min. 0.125 µg/mL PE-conjugated anti-CD45 mAb (clone 2D1, BioLegend, San Diego, CA, USA) was used to remove any contaminating hematopoietic cells and a 30nM SYTOX Green Nucleic Acid Stain solution (Thermo Fisher Scientific) was used for dead-cell staining. The top 5% of MUC16-positive cells and the bottom 30% of MUC16-negative cells were isolated using a FACSAria II cell sorter (BD Biosciences, San Jose, CA, USA). The data were analyzed using FlowJo (BD Biosciences) software.

### 4.10. Statistical Analysis

Paired *t*-tests were performed to compare two groups and Dunnett’s multiple comparisons tests were performed to compare experimental groups against a control group using the Prism 8 software (GraphPad Software, San Diego, CA, USA). The data are presented as mean ± standard deviation (SD) values derived from five or more independent experiments. * *p* < 0.05, ** *p* < 0.01, *** *p* < 0.001, **** *p* < 0.0001.

## Figures and Tables

**Table 1:** Downregulated genes with reduced 5hmC levels in *TET2* knockdown cells.

Downregulated genes with reduced 5hmC peaks in Exon regions in *TET2* KD cells									
MYEOV	BEST1	COLCA1	CREBBP	KRT34	FIBCD1	TNNI2	PLPP2	TLCD1	PLCB3
TMC5	TIMM44	SLC4A2	FAM107A	CARD10	PDXP	MIB2	TET3	SNX8	TNFSF14
TEAD4	NFATC4	KRT23	FASN	CFD	SEMA3B	LSP1	ATAD3C	CCDC88B	CAPS
COX4I2	MED24	ATP2A1	TNK2	ENO1	WNK2	MIDN	IFI27	TXNRD2	EMILIN2
CAPN12	PLEKHB1	DOCK6	MUC4	UPP1	P4HTM	SERPINF1	PTPRH	MSI1	NDUFA4L2
ZNF768	ATG16L2	MAL	B4GALT5	SPTBN2	KLF16	SLC9A1	LEFTY1	MB	C15orf39
KLK13	EPHB6	WTIP	TMEM205	DOT1L	QRICH2	ISYNA1	CHD1	HGD	RASGRP2
HES6	TNFSF13	MKNK2	REC8	MYLPF	FBXL19	REEP6	RNF223	MLPH	CCK
TBC1D1	LTC4S	SIGIRR	ZNF652	FUT4	PALM	H19	ARRB2	MSI2	TF
SLC7A1	ABCA7								
Downregulated genes with reduced 5hmC peaks in Intron regions in *TET2* KD cells									
ATP6V1B1	AGAP1	INF2	PALM	B4GALT5	NCMAP	ADIPOR2	MLPH	PPARGC1B	WNK2
KCNQ1	PPP1R9B	CARD10	DOT1L	SLC6A17	BFSP2-AS1	ABCA7	REEP6	SSBP3	SMDT1
CORO2A	SIGIRR	TEAD4	NTF3	MSI2	CCDC57	TNK2	SLC4A2	BGN	PRKCB
LYPD5	MUC4	SNX8	FAM20A	LMTK3	TXNRD2	TJP3	CSK	PHLPP1	JDP2
TMC5	EMILIN2	PKN1	ATAD3C	ASPHD2	WTIP	LSP1	SLC29A2	EMP1	PRIM1
ZNF652	TIMP2	MIDN	SEC1P	TET3	HYAL1	LYPD2	SH2D4A	BICDL1	SGF29
FAM3D-AS1	MKNK2	SERPINF1	PLPP2	LEFTY1	PLCB3	SLC16A3	CAPN12	PTPRH	MAL
GMPR	SPTBN2	ENO1	SLC9A3R2	FAM107B	SLC12A5	HEXB	R3HDM2	SLC9A1	SLC25A37
ATG16L2	NDUFA4L2	VWF	ECI1	CREBBP	KRT23	TNFSF14	MUC16	MIB2	WNT3A
EPHA8	SNHG17	KRT8	FASN	S100P	CAPNS1	SH3GL3	ATP2A1	MED24	MROH6
FBP1	RAB26	KRT78	UPP1	APOL6	IFI27	TLCD1	NQO2	PTPRD	BEST1
SIX1	CYBA	MOCOS	RNF223	EEF1A2	DGUOK-AS1	TBC1D1	SLC6A8	FAM120A	ARRB2
MRPL12	CRMP1	FIBCD1	FBXL19	P4HTM	TMCO4	TIMM44	PPP2CB	CRIP1	VEGFA
TMEM205	MCRIP2	MT1M	TMEM164	RASGRP2	MSI1	C15orf39	MAP4K1	KLK13	N4BP3
KLF16	VKORC1	LGALS9C	LEMD1	ALMS1	TF	SLC7A1	RASA4B	FAM3D	NXPH4
MYEOV	TET2	LRRC56	DCTPP1	LGALS9	ABCA4	WNT6	SGMS1-AS1	PDXP	DUSP5
H19	IZUMO4								
Downregulated genes with reduced 5hmC peaks in Promoter regions in *TET2* KD cells									
INF2	HGD	PRR34-AS1	B4GALT5	EIF4EBP3	CYSRT1	LSP1	KRT78	SERPINF1	LYPD2
KRT8	RAB26	TLCD1	NQO2	ARRB2	FAM107A	SPTBN2	PTPRH	CSK	NAT14
TJP3	SLC4A2	LEFTY1	PCYT2	MLPH	TMCO4	LGALS9C	TMEM205	CAPS	UPP1
IFI27	MAL	LRRC56	MYEOV	CCDC88B	JDP2	UBXN10	S100P	SGMS1-AS1	N4BP3
GMPR	CARD10	RHBDL1	IZUMO4	SMDT1					

## Data Availability

The RNA-seq data were deposited to the Gene Expression Omnibus under accession number GSE216213.

## Supplementary Materials

The following supporting information can be downloaded at: https://www.mdpi.com/article/10.3390/ijms24032841/s1.
