# Supplementary figures and images for: Epigenetic Regulation of Corneal Epithelial Differentiation by TET2
Source: Int J Mol Sci. 2023 Feb 2;24(3):2841. doi: 10.3390/ijms24032841 (PMC9917645; doi:10.3390/ijms24032841)

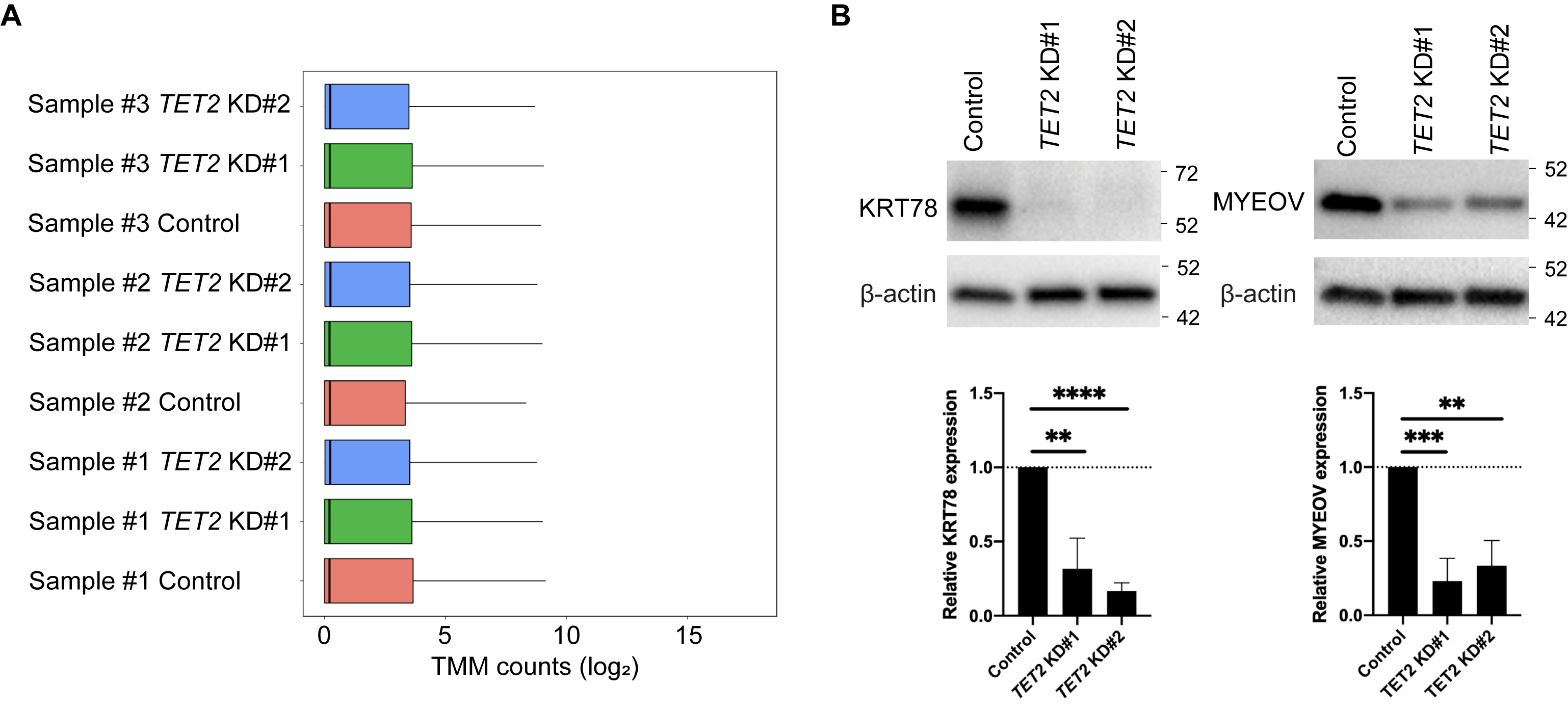

Supplement: Supplementary file 1 [file ijms-24-02841-s001.zip › TET2 Supplementary Figure 1 Final.tiff]
